# Development, Co‐Design and Pilot Testing of Resources to Support Informed Decision‐Making for Lung Cancer Screening in Australia: A Study Engaging Diverse Communities and Expert Healthcare Professionals

**DOI:** 10.1111/hex.70824

**Published:** 2026-08-18

**Authors:** Rachael H. Dodd, Kathleen McFadden, Marianne Weber, Dana Mouwad, Mei Ling Yap, Michelle Dickson, Simone Sheriff, Emily Stone, Dipti Zachariah, Alicia King, Nicole M. Rankin

**Affiliations:** ^1^ The Daffodil Centre The University of Sydney, and Cancer Council NSW Sydney Australia; ^2^ Sydney Health Literacy Lab, School of Public Health, Faculty of Medicine and Health The University of Sydney Sydney Australia; ^3^ Cancer Elimination Collaboration, School of Public Health, Faculty of Medicine and Health The University of Sydney Sydney Australia; ^4^ Statewide Health Literacy Hub NSW Health Sydney Australia; ^5^ The George Institute for Global Health, UNSW Sydney, Randwick Sydney Australia; ^6^ The Collaboration for Cancer Outcomes, Research and Evaluation (CCORE) Ingham Institute for Applied Medical Research, Liverpool Sydney Australia; ^7^ Liverpool and Macarthur Cancer Therapy Centres Western Sydney University, Campbelltown, NSW Sydney Australia; ^8^ The Poche Centre for Indigenous Health The University of Sydney Sydney Australia; ^9^ Department of Thoracic Medicine and Lung Transplantation St Vincent's Hospital Sydney Australia; ^10^ School of Clinical Medicine Faculty of Medicine and Health, UNSW Sydney Australia; ^11^ Multicultural Health Western Sydney Local Health District Sydney Australia; ^12^ Cancer Council Western Australia Subiaco WA Australia; ^13^ Evaluation & Implementation Science Unit, Melbourne School of Population and Global Health The University of Melbourne Melbourne Australia; ^14^ School of Public Health, Faculty of Medicine and Health The University of Sydney Sydney Australia

**Keywords:** co‐design, decision tools, end‐users, lung cancer screening

## Abstract

**Introduction:**

Australia's National Lung Cancer Screening (LCS) Program commenced in July 2025, targeting people at high risk of lung cancer based on age and smoking history. This study aimed to co‐design evidence‐based decision support tools to facilitate shared decision‐making (SDM) for LCS in collaboration with intended end‐users.

**Methods:**

Co‐design workshops were conducted with consumers, including Aboriginal and Torres Strait Islander peoples (4 workshops; *n* = 54), and clinicians/experts (2 workshops; *n* = 7). Participants were recruited through an independent social research company, an ongoing wellbeing support group, an Aboriginal Community Controlled Health Service and purposive sampling. Sessions involved structured activities to identify key content priorities and preferred formats for decision support tools. Workshop data were analysed concerning preferences regarding the key priorities for content and design of the tools. Cognitive interviews (*n* = 15) with an independent group of consumers were undertaken to test comprehensibility and refine design elements. Interviews were analysed against the key content areas that needed to be improved and the views of participants across the three tools developed. For each participant, their likes, dislikes, suggested changes, and how they compared across the three resources were documented. All resources were developed iteratively in alignment with International Patient Decision Aid Standards.

**Results:**

Key shared priorities across consumers and clinicians included information about the screening process, what happens after the scan, benefits of screening, the need to manage cancer and smoking‐related stigma and discrimination, tailoring for different language groups, and cultural safety. Consumers emphasised the need for visual representations of the screening pathway and testimonials from previous participants, while finding icon arrays confusing and overly statistical. Both groups highlighted the importance of supporting engagement with screening and reframing potential harms as ‘other considerations’ rather than deterrents. Feedback from cognitive interviews informed refinements to improve clarity and usability. The final suite of resources comprised a 16‐page A5 booklet, a 2‐page A4 leaflet, and a 3‐min animated video.

**Conclusion:**

This co‐design process produced three decision support tools to support informed choice within Australia's National LCS Program. The tools were found to be clear, acceptable, and adaptable, with potential for broader use in international LCS implementation.

**Patient or Public contribution:**

An Aboriginal Reference Group of seven members was formed at project inception and provided cultural oversight and support in the design and conduct of the research with Aboriginal and Torres Strait Islander peoples. Members of the public who might be eligible or may have a family member eligible for lung cancer screening, were included in the conduct of the research.

## 1 Introduction

Lung cancer is the leading cause of cancer death worldwide, with most diagnoses occurring at an advanced, incurable stage [1]. In Australia, lung cancer accounted for an estimated 8,994 deaths in 2025, representing 16.8% of all cancer deaths [2]. Mortality and burden of lung cancer disproportionately affects people in regional and remote areas, lower socioeconomic groups, culturally and linguistically diverse communities, and Aboriginal and Torres Strait Islander peoples [3]. Lung cancer screening (LCS) via low‐dose computed tomography (LDCT) has the potential to save thousands of lives through early detection and treatment [4]. The National Lung Cancer Screening Program in Australia targets 50–70 year olds, who currently smoke cigarettes or have quit within the last 10 years, and who have smoked at least 30 pack‐years in their lifetime (e.g., a pack a day for 30 years, or 2 packs a day for 15 years) [5]. Costs for the primary and follow‐up scans are covered by the Medicare Benefits Schedule within Australia's universal healthcare system.

However, screening also has downsides, such as false positives, and potential for unnecessary procedures, overdiagnosis, and psychological burden [6]. As participation decisions may vary according to individual values and preferences, shared decision‐making (SDM) is an important component of best‐practice patient‐centred care and is mandated for LCS in the USA [7]. SDM is a collaborative process that integrates the best available evidence with a person's values, goals and healthcare preferences [8]. Decision support tools – including pamphlets, videos, and web‐based resources – are commonly used to support SDM by helping people understand available options and consider how these align with their preferences. Developing these tools with the people who will use them, to ensure they are understandable, balanced and usable, is essential to successful implementation [9]. The International Patient Decision Aid Standards (IPDAS) Collaboration has developed an evidence‐informed framework to guide the development of these tools [10]. In LCS, decision support tools have been shown to improve knowledge and informed choice, reduce decisional conflict [11], and are acceptable and feasible for use in practice [12].

Existing decision support tools for LCS have been designed and tested primarily in the USA [11], where LCS was implemented in 2013. Tools designed specifically for the Australian context are needed given the differences in priority populations, healthcare system and landscape, and LCS service design. To date, the only published LCS decision support tool for use in Australia was designed in 2018 prior to the announcement of a national LCS programme [13]. Key programme details are different to those included in the existing tool (e.g., use of categorical eligibility criteria rather than risk prediction score eligibility criteria). With the commencement of LCS in Australia in July 2025, there was immediate need for decision support tools to enable rapid translation of SDM into practice. In addition, from a systems perspective, LCS is only effective and cost‐effective if the right people participate. SDM can support this by (1) helping engage underserved priority groups in LCS discussions (also reducing health inequities) [14, 15]; and (2) helping target screening toward those most likely to benefit, while discouraging screening among those least likely to benefit [16]. The aim of this study was to co‐design resources to support informed choice in LCS in Australia.

## 2 Methods

**Figure 1:**
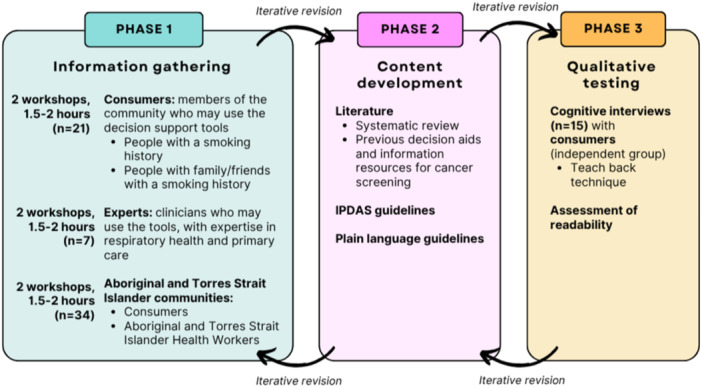
Co‐design and development procedures. *Note:* Arrows indicate ongoing iterative development between phases (e.g., following qualitative testing, content development was revisited to incorporate feedback). IPDAS, International Patient Decision Aid Standards.

This study employed a co‐design and development methodology, broadly following the systematic co‐development approach described for producing decision aids [17]. The content and development process of the decision support tools aligned with the IPDAS criteria [18]. Development involved three phases of 1) information gathering, 2) content development, and 3) qualitative testing (Figure 1). This figure demonstrates the iterative co‐design process with phases 1 (workshops) and 2 (literature, IPDAS and plain language guidelines) contributing to the development of the resources and phase 3, the cognitive interviews. Ethics approval was awarded by Western Sydney Local Health District Human Research Ethics Committee (2022_ETH00375) and the Aboriginal Health and Medical Research Council Human Research Ethics Committee (1961/22). The study findings were reported in accordance with the COnsolidated criteria for REporting Qualitative research (COREQ) checklist [19] (Appendix 1).

To guide research activities with Aboriginal and Torres Strait Islander peoples, an Aboriginal Reference Group (ARG) was formed at project inception. Terms of Reference were developed in collaboration with the ARG, detailing the role of the group in providing cultural oversight and support in the design and conduct of the research. Members of the ARG (*n* = 7) mostly worked in research or advocacy roles and provided guidance on the study design and the conduct of workshops with Aboriginal and Torres Strait Islander peoples.

### 2.1 Phase 1: Information Gathering

Co‐design of the resources followed the New South Wales (NSW) Health Agency for Clinical Innovation iterative model of co‐design—engage, gather, understand and improve [20]. Co‐design was selected to incorporate the complementary expertise of people who may be eligible for screening, their wider community, and clinicians. This ensures the tool content and format are relevant, while enabling iterative refinement through stakeholder feedback.

Initial workshops were to determine (1) the information consumers and clinicians felt was most important for deciding about LCS, and (2) how the information should be presented.

*Consumer workshops*. These involved a series of workshops (each 1.5–2 h) with consumers in March and June 2023, defined as people who may use decision support tools for themselves or a friend/family member to take part in an Australian National LCS Program. This included (1) those with any smoking history, and (2) those with family/friends with a smoking history (given social influence of friends/family can be instrumental in screening decisions) [21, 22]. Consumer participants in the first co‐design workshops were recruited via Taverner Research Group, an independent social research company who recruit using random digit dialling by defined postcode area. Taverner Research Group have access to list‐based calling where age and gender is known, which can be used, if needed, to help recruit specific participants. They also use social media methods to recruit including Facebook and Instagram. We purposefully used quotas to target people from culturally and linguistically diverse backgrounds (using speaking a language other than English at home as a proxy measure). In addition to this, further strategies were undertaken in partnership with Multicultural Health Services (Western Sydney Local Health District) to strengthen cultural safety and inclusion. As part of this collaboration, cultural expertise was provided to ensure that co‐design workshops and decision support tools were culturally safe, inclusive, and relevant for people from culturally and linguistically diverse communities. Workshop content was reviewed through both a multicultural health and health literacy lens, with potential cultural sensitivities, language barriers, and areas requiring clearer communication identified and addressed. Targeted recruitment strategies were implemented to maximise culturally and linguistically diverse participation, drawing on established community networks.

Two co‐design workshops were also conducted with participants in regional areas of Australia in Aboriginal and Torres Strait Islander communities, in February and April 2024. Participants for these workshops were recruited through an ongoing wellbeing support group and an Aboriginal Community Controlled Health Service. All participants provided informed written consent.

*Expert/clinician workshops*. Separate workshops were conducted with clinical experts (termed the steering committee) in March and August 2023, to engage, gather and understand key priorities to include in decision support tools. Expert participants were purposefully recruited from the research teams' networks, and consisted of 7 members: 4 respiratory physicians, a primary care specialist, a behavioural scientist and a consumer representative.

Workshops were conducted in‐person and via videoconference, led by a member of the research team (R.D. and N.R.) both with PhDs, or an external facilitator. Facilitators were all female and had experience in conducting workshops or focus groups with consumers on public health topics for the purpose of research and had an interest in cancer screening. Facilitators introduced themselves and the team at the start of the workshops and the aims of the research. All workshops were co‐facilitated with 1‐3 other study team members. Content initially focused on (1) co‐design and the purpose of the project, (2) lung cancer screening, and benefits, harms, and considerations for screening, and (3) shared decision‐making (SDM) and decision support tools. Given the limited familiarity with SDM observed in the first consumer workshop, subsequent workshops included additional content explaining the concept. These sessions also incorporated an interactive discussion in which participants were invited to consider the potential benefits and harms of LCS for different individuals and to reflect on whether screening would be worthwhile for each person. Later workshops were focused on development of the decision support tools, which were iteratively revised according to feedback from both groups. A detailed overview of workshop content and timeline is provided in Appendix 2. Workshops included a mixture of presentations (via Powerpoint), both whole and smaller group discussions, and idea generation activities.

All qualitative data were collected through audio recording, with transcription via an automated tool (Otter.ai). Detailed field notes were made throughout the discussion, and participants were always offered the opportunity to provide feedback via email. Results of workshops and interviews were discussed at regular intervals among the research team, with consensus on key themes and outcomes from the data driving iterative revision of the decision support tools.

Qualitative data were analysed by two coders (R.D. and K.M.). The coders read the transcripts of the workshops to familiarise themselves with the data and grouped the quotes concerning preferences regarding the key priorities for content and design of the tools, and understanding of shared decision‐making. The two coders discussed their findings and resolved differences. The analysis informed the second phase of content development.

### 2.2 Phase 2: Content Development

Based on the findings from the first workshops, a published systematic review [23], previous decision support tools [13, 24, 25] and information resources for cancer screening, three decision support tools were developed. These were designed using IPDAS standards (Appendix 3) [26]. The content was initially developed by the lead author (R.D.) and then reviewed and refined by the research team. The quantitative outcomes were developed using evidence from lung cancer screening trials and studies (MW) [27, 28, 29, 30, 31, 32].

Plain language guidelines [33] were followed in developing the content: using active voice, using common rather than complex words, using a positive tone when possible, writing for the reader and keeping words and sentences short. Readability of the resources was also checked using the Sydney Health Literacy Editor [34] and adapted as needed.

We engaged an expert graphic designer to develop an attractive product that facilitated easy navigation through the text.

### 2.3 Phase 3: Qualitative Testing

Individual cognitive interviews were conducted in September and October 2023 with a different group of consumers to assess comprehension of key content and preferences regarding presentation, recruited using Taverner Research Group. These interviews used a standard teach‐back technique [35], asking participants to describe in their own words what selected parts of the decision support tools were trying to communicate. These individual cognitive interviews with consumers were to test and improve the resources. Interviews lasted an average of 50 min.

Two members of the team read through the transcripts and identified key content areas that needed to be improved and the views of participants across the three tools developed. For each participant, their likes, dislikes, suggested changes, and how they compared across the three resources were documented. In presentation of results, supporting quotations have been edited minimally for comprehension.

Following each workshop and cognitive interview round, feedback was collated and reviewed by the research team. Suggested modifications were assessed according to relevance to the tool's purpose, consistency with shared decision‐making principles, recurrence across participants, and feasibility of implementation.

Data was triangulated across all the workshops (culturally and linguistically diverse, Aboriginal and Torres Strait Islander peoples, steering committee) and the cognitive interviews, with no further themes raised in the cognitive interviews, indicating data saturation was achieved.

## 3 Results

### 3.1 Overview of the Decision Support Tools

Twenty‐one consumers were involved in the first of two workshops held in Western Sydney, New South Wales (NSW) in March 2023 and 16 consumers in June 2023. Sixteen participants (5 consumers and 11 Aboriginal Health Workers/Practitioners) took part in a workshop held in Wagga Wagga, NSW in February 2024 and seventeen (13 consumers and 4 Aboriginal Health Workers/Practitioners in Aboriginal Health) in a workshop in Dubbo, NSW in April 2024. Seven members of the expert steering committee took part in workshops in March and August 2023. The main motivators for participation in the study included having friends/family members who smoke or have died from lung cancer, being intrigued by lung cancer screening, appreciating the opportunity for their voice to be heard, believing engagement is important, and being able to address false beliefs about lung cancer in their family. Participants characteristics are described in Table 1.

**Table 1:** Participant characteristics.

	Phase 1		Phase 3
	Attended both workshops (*n *= 16)	Attended workshop 1 only (*n* = 5)	Cognitive interviews (*n* = 15)
Age			
18–29	3	1	3
30–39	3	2	4
40–49	2		3
50–59	3	1	3
60–69	4	1	1
70–79	1		1
Gender			
Female	8	2	8
Male	8	3	7
Working status			
Retired	3	1	2
Working part‐time	3		3
Working full‐time	7	3	6
Working casually	1	1	
Studying	1		1
Stay at home parent/Carer	1		1
Pension			1
Self‐employed			1
Education			
School certificate (year 10 or equivalent)	1		1
High school (year 12 equivalent)	1		5
Diploma	4		
Trade certificate	2		1
Undergraduate degree	6	3	5
Postgraduate degree	3	1	3
Language spoken at home			
I speak English only	9	3	8
I speak English and another language at home	7	2	7
Mandarin	2		1
Cantonese	2	1	1
Bahasa Indonesian	1		
Russian		1	
Armenian	1		
Telugu	1		1
Hindi			1
Greek			1
Croatian			1
Tamil			1
Italian			1
Smoking status			
Is currently smoking	5		2
Used to smoke	4	4	6
Never smoked	7	1	7
Smoking status of family/friends			
A close family member or friend currently smokes	12	3	8
A close family member or friend used to smoke	3	2	6
No close family or friends has smoked	1		1
Experience with lung cancer			
A close family member or friend has experienced lung cancer	8	2	6
No close family member or friend has experienced lung cancer	8	3	9

Aboriginal and Torres Strait Islander community workshops	Phase 1	
	Dubbo (*n* = 17)	Wagga Wagga (*n* = 15)*
Age (mean [SD] years)	68.7 [9.9]	45.6 [7.3]
Age range	50–82 years	28–72 years
Gender		
Female	13	11
Male	4	4
Indigenous status		
Aboriginal and/or Torres Strait origin (not specified)	10	9
Aboriginal origin	5	5
Torres Strait Islander origin		
Both Aboriginal and Torres Strait Islander origin		
Neither Aboriginal nor Torres Strait Islander origin	2	1
Language spoken at home		
English is the main language spoken at home	17	15
Smoking status		
Has a smoking history (not specified)	5	8
Is currently smoking		3
Used to smoke		1
Never smoked	12	2
Not answered		1
Personal or family experience with lung cancer		
Personal history of lung cancer		
Family history of lung cancer	5	3
No personal or family history of lung cancer	11	11
Not answered	1	1

*Data from one participant missing.

### 3.2 Phase 1: Information Gathering

The first workshops with consumers and the steering committee determined the key priorities to include in the decision support tools for deciding about participation in LCS. The workshop participants wanted to know the benefits of LCS and gave suggestions for how information should be presented. This included having visual representation where possible, using photos rather than drawings, and ensuring that graphs were simple and easy to interpret, with minimal focus on statistics. Information was to be short, sharp and encouraging to ‘get people in the door’ for LCS.

Participants also talked about the need for the role of family and friends to help break down barriers around cancer and smoking related stigma and shame that may stop or delay participation in LCS. All participants flagged the important role of family and friends in screening decisions, particularly children of those in the eligible age‐range (50–70 years old in Australia), and stressed the importance of engaging family members in the decision‐making process. Aboriginal and Torres Strait Islander participants explained that the whole family is taken along a cancer journey, therefore the resources should support potential participants in explaining screening to family members, as many people ‘don't like to talk about cancer’. Across all workshops, participants identified that use of the word ‘cancer’ created fear. Participants overwhelmingly preferred ‘lung screening’ or ‘lung check’ (even before discussion of the UK's Targeted ‘Lung Health Check’ nomenclature) [36]. Format suggestions were primarily around information being sharable (e.g. via social media), where a person's social network (e.g., children or carers) can send to people they know who might be eligible.

There were some additional considerations for Aboriginal and Torres Strait Islander peoples that came from the workshops held in regional NSW. They suggested leveraging the annual Health Check (Medicare Benefits Schedule Item 715) that is currently available to all people of Aboriginal and Torres Strait Islander descent to talk about LCS, given one of the purposes is to support participation in population health programmes (e.g., vaccination, cancer screening). Participants flagged throughout consultation that the LCS approach for Aboriginal and Torres Strait Islander people's needs to be designed specifically to meet community needs. Aboriginal and Torres Strait Islander Health Workers identified that a LCS decision may be a burden, adding ‘another thing’ for people to consider; some thought that it should be included as part of other health initiatives to reduce potential decisional fatigue. This was a similar sentiment to other workshops which thought ‘getting people in the door’ was the most critical issue. There was also mention of mistrust of information on social media, even information from government or official pages. Participants said that they relied on a doctor/specialist explaining information and that the most trusted sources are Aboriginal and Torres Strait Islander channels – e.g., Aboriginal Community Controlled Health Service, not just state health social media channels.

**Figure 2:**
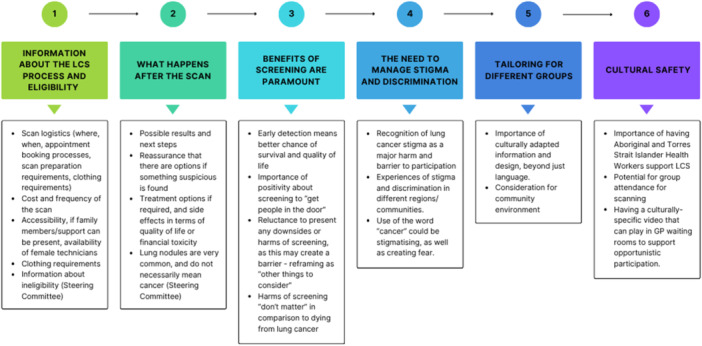
Key priorities for end‐users from co‐design workshops. LCS, lung cancer screening.

Figure 2 presents six major domains across the screening pathway: information about the process, what happens after the scan, benefits of screening, the need to manage stigma and discrimination, tailoring for different language groups, and cultural safety. Each domain highlights specific informational needs and considerations influencing engagement with LDCT screening.

Appendix 4 summarises major priority areas, detailed informational needs within each domain, and illustrative supporting quotes from participants. Priorities include understanding the LDCT scan process, post‐scan outcomes, perceived benefits of screening, stigma and discrimination, cultural and linguistic considerations, and the importance of culturally safe care. Quotes are presented verbatim to reflect participant perspectives and highlight contextual factors influencing screening engagement.

#### 3.2.1 Design Considerations

Participants wanted to see a visual representation of the LCS process, testimonials, narratives and/or quotes from previous LCS participants.

The testimony was the strongest part, so it actually showed the process, the alleviators of fear … And then the fact it was only in hospital for two days and then he's got a follow up, he's going to six months and then you know he's fine.

(Consumer)

Word of mouth was described as the main information distribution method in Aboriginal and Torres Strait Islander communities. Therefore, having testimonials of people who have been through it themselves was seen as very important. Workshop participants described that in previous population health campaigns, the use of local Aboriginal designed T‐shirts was effective in creating conversation and as an incentive to participate. Good rapport with a specific worker or doctor who has respect from community was also reported as important.

Most consumer end‐users reported that icon arrays were too statistics‐focussed and difficult to comprehend. The steering committee agreed that in their experience icon arrays were unhelpful for risk comprehension. Participants in the rural workshop noted ‘*I'm just saying the way a rural person would read this is different to a person that lives in Sydney. Out of 1000 is no big deal in Sydney, but if you live in a rural area, that number looks different*’. (Consumer)

Participants made suggestions about the design and the content, noting the importance of keeping it simple, precise, short, encouraging, and interactive, with use of infographics to reduce the amount of text. The setting that the information was to be used in was a careful consideration; having the information available in the GP office was deemed useful.

Yeah, I think flyers are really good … when you're in a GP or something, a lot of them have those little boards with stacks of different flyers. And I pick them up and read them. Even if I don't think they're relevant to me, just to kill time … they're a good opportunity to raise awareness where people may not otherwise think about these sorts of things … they look personalised as well.

(Consumer)

Participants were not interested in a dedicated LCS app, as they advised that apps are for long‐term use, and they would only use it a handful of times for LCS. Instead, it was suggested that there could be some information about LCS integrated into existing apps (e.g. ServiceNSW which is used to access other government services) may be useful.

I'm not going to work up space and resources on my phone [for] something that is maybe gonna be used once or twice.

(Consumer)

Having tiers of information with different levels of detail was also discussed, from high level overviews to resources with more detail.

#### 3.2.2 Understanding of SDM

The concept of SDM was introduced during presentation activities in initial workshops. For many participants, SDM was a new concept to grasp, as their experience was that health professionals made decisions based on their clinical judgement with no input from them. Participants familiar with SDM mostly reported this from the angle of treatment decisions, therefore framing screening or early detection as a decision that should be shared was a new concept.

Following presentation and activities, participants understood and appreciated the role and value of SDM, and how it differed from usual clinical practice. They also recognised that people require appropriate information about medical decisions to make the right choice for them, and that decisions can often be made with others such as caregivers or others in their community.

Best case scenario, I suppose, is that the patient, family and the health professional are making decisions together.

(Consumer)

The concept behind this is that as a patient, you can choose how much you want to be involved in that decision, it's really, really important for the doctor and the patient to work together … some doctors that can be very dismissive or they don't give you all the options.

(Consumer)

It was also recognised that doctors need to be able to communicate with diverse populations or ideally be from the same community, and that it is the role of the whole care team to aid in decision‐making. There were concerns about the ability to implement SDM and the consequences for the general practitioner (GP) if a person decides against best practice. Quality over quantity of life was valued by participants.

One participant also gave a personal story, including the role of family and carers in supporting shared decision‐making:

Is that my grandfather, who spoke very little English when he was diagnosed with lung cancer, and he was told the options, he said he didn't want to go through the chemotherapy because, in his experience, it's a lot of pain, and he didn't want to put that on the family, because in our culture, in most ethnic cultures, the family looks after the ill person, and he feels very strong headed and said, ‘No, I'm not going down that road’. So we had to take some time to get him to understand the benefits. He ended up getting two extra years, which he was thankful for, but if we had just left that decision to him… I'm talking from a cultural perspective …and I'm sure every ethnic… it's a family decision, and I'm a granddaughter, and I was still part of it. So to understand that it's not just the patient you're talking to in these kind of communities … because we're the carers.

(Consumer)

#### 3.2.3 Phase 2: Content Development

Participants suggested the need for ‘tiered’ levels of information – i.e., different formats which provide different levels of detail. Based on this, three decision support tools were developed as prototypes: a 16‐page A5 booklet, 2‐page A4 leaflet and a 3‐min animated video. Having multiple options of accessing the decision support tools were important, such as online, and talking to someone in person. Table 2 shows the content of each tool developed.

**Table 2:** Content included across the three tools.

Tool	Content
16 page A5 booklet: statistics tables	The booklet contained information about the aim of the tool, information about lung cancer, lung cancer screening, eligibility criteria and risk assessment, what screening involves, the benefits and harms of screening (over 2 pages), possible scan results, and lung cancer treatment. The booklet also contained a tool to help calculate pack years, a testimonial and summary statistics in a table comparing health outcomes of screening versus no screening. A short values‐based questionnaire was also included, along with a page to write notes.
16 page A5 booklet: icon arrays	As above, but with the statistics displayed as icon arrays (based on literature suggesting that icon arrays support numerical risk comprehension). [37, 38, 39]
2‐page leaflet	The leaflet contained all the information from the longer 16‐page booklet, but with less detail on the benefits and harms, no testimonial and no area to write notes.
3 min video (animated, 3 min, 23 s)	The video included most of the information from the 16‐page booklet, with animations, subtitles and narration, excluding information on eligibility criteria, risk assessment or an explanation of pack‐years. A professional animator developed the video based on a script and reference videos provided by the lead researcher (full video available here: https://www.youtube.com/watch?v=dTteUpo1GhY)

Each tool emphasised that screening is a choice and was framed as a resource providing information to support participants in choosing whether to have screening or not. The longer versions included the use of a testimonial as this was a highlight for consumers. Two versions of the booklet were developed – one with numerical information displayed with icon arrays and the second using percentages in a table to facilitate comparison between the options (screening vs. not screening).

#### 3.2.4 Feedback on Initially Designed Tools

In general, participants were very positive about initial design of the three tools. Specific feedback was given across the three resources. While many suggestions resulted in wording, layout, and content refinements, not all participant recommendations were incorporated. Suggestions were less likely to be actioned where they reflected isolated preferences, conflicted with the tool's intent to support shared decision‐making, or would have reduced balanced presentation of options.

*Booklet*. Some of the participants expressed that there was a lot of information in the booklet and that it was a ‘bit too text‐heavy’ and recognised that there seemed to be a few different purposes of the booklet.

So I thought the whole document, in general, is a bit too text‐heavy and a bit confusing, confusing in the sense of, is it a qualifying document? In the sense that you're trying to qualify people, whether they should be screened? Is it informational? One when you've got things like information on one cancer and then you even got a survey on the back? So there's tends to be a number of purposes for this one document.

(Consumer)

Participants liked the infographics and the interactive parts, with the need for some parts to be better balanced and clarified.

Some parts need adjustments and some little points of clarification in your text, like sometimes they use acronyms that need to be removed.

(Consumer)

The values clarification activity at the back of the booklet was liked and appreciated, seen to make it interactive as well as facilitating the joint discussion with the healthcare professionals.

Love this, where you got to do a self‐analysis, it makes it somewhat interactive. And I think based on your selections, it's also it's a good opportunity to be part of that.

(Consumer)

*Two‐page leaflet.* Despite being shorter than the booklet, this resource was viewed as having all the necessary information in terms of the process of screening, benefits and harms. It was also seen as balanced and factual.

Firstly, I had to identify whether or not it was relevant to me based on my smoking history and age. It went through the stats and the pros and cons that you may experience, and then the last little survey interactive part was really more of a point to weigh out and balance it. Who was going to come and help me balance whether or not this was right for me? So I actually saw it as a good one pager, really, to take me on the journey in a simple way.

(Consumer)

One participant explained how the numbers given help put things into context and help to make an informed decision.

Like, when you put percentages up there, it really shows you a reality of what you do every day and the percentage of danger, like crossing the road wearing high heel shoes, like, we don't think about those sort of things, but everything has a factor of risk, so putting percentages on it just straight away gives you the clear numbers, and then you can make an informed decision, because that's what it's about.

(Consumer)

Some suggestions were made to the summary table, with the thought that the points were not balanced between positives and negatives.

If we had two points for positive and two points for negative, that might be a way to do it … people are more confident information is when you have a balanced condition. So if you already provide a balance, it will be that information will be perceived as credible.

(Consumer)

The interactive nature of the one‐page summary was appreciated, but concerns were raised about the complexity of terms like ‘pack years.’

*Video.* Participants found the video format to be easy to understand and engaging, and overall preferred it compared to the paper resources. Participants expressed that the benefits were just touched on at the start, but the rest of the video was skewed towards harms, and more balance was needed. There were different or conflicting views about the animation style; some people wanted to add different animation features or use different styles. There were some technical suggestions on things to add in or take out in terms of the information presented and pictures and the voice‐over used.

I think would be to keep the message sort of simple. I think you're giving the consumers a little bit too much information, too much credit [for the] decision process. I think if we're told to do something, and for our benefit, we'll do it. So it's a matter of sort of accentuating those positives and reducing those negatives.

(Consumer)

#### 3.2.5 Phase 3: Qualitative Testing Using Cognitive Interviews

Fifteen consumer participants took part in cognitive interviews (see Table 1). Overall, all resources were well received by participants, with minor suggestions for changes that were actioned (see Table 3).

**Table 3:** Suggested changes that were actioned as a result of the cognitive interviews.

Suggested changes	Overall impressions across the three tools
Eligibility Make it clear that all 3 criteria need to be met, move it earlier, make ‘quit within 10 years’ more obvious Pack years Make sure pack year calculator is in all resources, make it clear the 30 pack years is linked to eligibility criteria (booklet) and clarify what it means Symptoms Include examples of symptoms and be clear screening is for people with no symptoms Results State how long to expect to wait for results, some distinction between normal and indeterminate for levels of concern, remove word ‘additional’ Treatment Move nearer to the back or on its own page, include chemotherapy and radiation and what ‘a mix’ of treatment is, clarify what a biopsy scan is and what other scans could be (video) Values table Instructions for completion and summary of what scores mean, make screening/no screening headers clearer Design Less busy, increase white space, include picture of the lungs and highlight chest in computed tomography (CT) scan, add logos, remove icon array/numbers for no screening, change colours of testimonial, hyperlink to sections from contents page, include screening steps in leaflet Language Just use CT scan, include prompts to encourage people to discuss with friends/GP, include subtitles for whole video, clarify ‘early‐stage cancer’ not ‘early stage of life’, clarify that low dose is about radiation, replace ‘disadvantages’ Referrals for the tools in other languages	Overall, participants like having multiple options which have use for different purposes Two‐page leaflet Awareness tool short and to the point, beneficial for those who have trouble reading and/or time poor and quick way to assess eligibility – all information is there, just more concise Information is overwhelming, with people needing to investigate details further Booklet Mix of opinions about amount of information, some liked having all the details, some preferred icon arrays/some without and liked summary page and testimonial How results are presented in this version as preferred Video Some points clearer in video version than written, important for those audio learners and those who can't read, seen as a starting point and would then look for more details, liked visuals, negatives of screening are more obvious

Readability assessments were conducted on the final three resources using the SHeLL Health Literacy Editor [34]. The 16‐page booklet with icon arrays had a readability grade of 9.1. Both the 16‐page booklet without icon arrays and the 2‐page leaflet had a readability grade of 9. The video script had a readability grade of 8.6.

## 4 Discussion

This paper describes the co‐design process of developing three decision support tools for LCS in Australia. To our knowledge, this is the first co‐design research project about LCS to be conducted with such diversity of communities in the Australian setting using a comprehensive methodological approach. Consumer participants, as well as an expert steering committee of clinicians, provided key content priorities and design preferences to include in decision support tools for LCS. Aboriginal and Torres Strait Islander peoples are disproportionately impacted by lung cancer and are a priority group for LCS in Australia. Additional workshops were undertaken with Aboriginal and Torres Strait Islander peoples, to respect distinct cultures, needs and priorities. Importantly, Aboriginal and Torres Strait Islander cultures are diverse, with unique traditions, languages, beliefs, and values, and further work is needed to tailor resources to the needs of different communities. Across all consultations, key priorities included many process‐driven and logistical points such as what happens at the scan and where to go. This aligned with the content of previously developed decision support tools for LCS [11]. Other key considerations included the impact of smoking and cancer‐related stigma and the need for the information to be culturally adapted and safe for different communities. The use of cognitive interviews enabled in‐depth testing of each of the tools, with detailed feedback and improvements iteratively implemented.

A key aspect of cultural adaptations and cultural safety was a strong sense of community. For all participants, the need to include family was key, as they play a large role in the decision‐making process for both the culturally and linguistically diverse populations as well as Aboriginal and Torres Strait Islander communities. Finding your way is a model which has been created specifically by and for Aboriginal people to make decisions together with their healthcare providers [40]. ‘Community, Family and Kinship’ is a key structure informing people's choices in this model, as are personal experiences, supporting the findings from these workshops to include testimonials of people and polo shirts to promote screening.

This research contributes to the broader literature aiming to develop information and shared decision‐making resources to ensure patient‐centred care. We used the participatory method of co‐design to develop decision support tools for a new cancer screening programme, involving three key steps with iterative revision: information gathering, content development and qualitative user testing. This research was particularly timely, with the resources developed ahead of the implementation of a National Lung Cancer Screening Program commencing in Australia. Not all decision support tools are developed using this participatory approach from the beginning, with many US based decision support tools developed first by researchers, with feedback and/or evaluation following with end users [41, 42, 43, 44, 45].

We identified a preference across the sample for the numerical information to be displayed in a table rather than icon arrays. Icon arrays are intended to visually display numerical information, representing the numerator and denominator together via differently coloured icons arranged in a matrix. The table enabled us to have consistency in information across the booklet and leaflet, as the numerical information in the table could fit in the 2‐page leaflet, whereas the icon arrays would not. ‘Option grids’, which present a brief summary of options in a table, have received positive feedback from patients in the past [46]. Importantly, not all participant suggestions were incorporated. Due to the aim of the resources being to support shared decision‐making, we did not adopt changes that would have made the content more prescriptive, reduced the visibility of options, or prioritised clinician‐directed recommendations over patient preference clarification. In this sense, the refinement process involved balancing responsiveness to user feedback with preservation of the conceptual integrity of the tool.

These three tools provided patients with more interactive ways to gain knowledge about LCS and it is the hope that these tools will enhance participants decisional quality, with previous research finding LCS completion and intention to complete, to be higher for those who used a decision aid [47]. Further research tested these tools in a randomised trial and found each of the three tools were effective in supporting informed choice [48]. Many of the decision aids developed in the US have been print, web or video based [11], reflecting those preferences of the end users in this study.

Given SDM for LCS is likely to be a new concept for many primary care providers, we followed one of the recommendations from Volk and Stacey to ensure high quality SDM for LCS by using the IPDAS checklist to minimise risk of bias to patients (see Appendix 3) [26]. Only five of the decision aids found in a systematic review of decision aids for lung cancer screening were developed using this checklist [11]. Another recommendation from the checklist is for health care professionals to undergo training in SDM, which could potentially be a recommendation in Australia given the commencement of a national targeted LCS program.

### 4.1 Strengths and Limitations

A key strength of this study is the co‐design approach, including all potential end‐users of a decision support tool (including family members of potential LCS participants), and experts. This has ensured that the tools developed were to meet the needs of potential participants and health care professionals. Our use of a three‐phase staged approach ensured the co‐design process was rigorous and iterative, and engaged perspectives from a large sample of stakeholders. The development of these resources also benefited from the inclusion of potential participants from priority populations with both design and cultural considerations. System‐level cultural expertise provided by Multicultural Health Services through existing trusted community networks maximised culturally and linguistically diverse participation and supported culturally safe discussions and meaningful engagement. We acknowledge that while culturally and linguistically diverse communities were included, workshops required English literacy and more work is needed to test tools in translated, culturally adapted versions.

A limitation of this study is that it was conducted prior to the development of the Australian National Lung Cancer Screening Program guidelines, where some elements of the screening and assessment pathway differed to the information presented in the SDM tools. However, the approach, design, and most of the LCS information included in the SDM tools remain relevant for the current program, and could be easily adapted. Consultations were limited to English‐speaking participants, including Aboriginal and Torres Strait Islander peoples and individuals from culturally and linguistically diverse backgrounds. As a result, perspectives of non‐English‐speaking populations who may face additional barriers to accessing and understanding LCS information were not captured, however, in‐language focus groups have documented informational preferences for LCS [49]. As only one general practitioner was included in the steering committee, the views of primary care may not have been fully captured. These will be explored in further research testing the decision support tools. While these tools provide a valuable starting point for supporting informed decision‐making in LCS, future research should focus on culturally adapting the resources through in‐depth consultation with Aboriginal and Torres Strait Islander peoples and culturally and linguistically diverse communities.

## 5 Conclusion

The use of the information gathered from workshops with consumers, healthcare providers and Aboriginal and Torres Strait Islander peoples enabled three decision support tools to be developed. Successful implementation of these resources into the National Lung Cancer Screening Program will increase informed choice and may increase participation, but require further adaptation with priority populations. These tools have now been adapted for use in the National Lung Cancer Screening Program.

## Author Contributions

**Rachael H. Dodd:** conceptualisation, investigation, funding acquisition, writing – original draft, methodology, formal analysis, supervision, validation, writing – review and editing. **Kathleen McFadden:** investigation, writing – original draft, methodology, data curation, formal analysis, project administration. **Marianne Weber:** writing – review and editing, methodology. **Dana Mouwad:** methodology, writing – review and editing. **Mei Ling Yap:** data curation, writing – review and editing, methodology. **Michelle Dickson:** writing – review and editing, data curation, methodology. **Simone Sheriff:** data curation, investigation, methodology. **Emily Stone:** writing – review and editing. **Dipti Zachariah:** writing – review and editing, data curation, methodology. **Alicia King:** writing – review and editing, data curation. **Nicole M. Rankin:** writing – review and editing, investigation, methodology, supervision, data curation.

## Conflicts of Interest

The authors declare no conflicts of interest.

## Data Availability

The data that support the findings of this study are available on request from the corresponding author. The data are not publicly available due to privacy or ethical restrictions.

## Appendix 1 COREQ Checklist

Consolidated criteria for reporting qualitative research (COREQ): 32‐item checklist

Developed from:

Tong A, Sainsbury P, Craig J. Consolidated criteria for reporting qualitative research (COREQ): a 32‐item checklist for interviews and focus groups. International Journal for Quality in Health Care. 2007. Volume 19, Number 6: pp. 349 – 357

**Table:**

Item No	Guide Questions/Description	Reported on Page #
*Domain 1: Research team and reflexivity*		
Personal Characteristics		
1. Interviewer/facilitator	Which author/s conducted the interview or focus group?	Page 6
2. Credentials	What were the researcher's credentials? E.g., PhD, MD	Page 6
3. Occupation	What was their occupation at the time of the study?	Page 6
4. Gender	Was the researcher male or female?	Page 6
5. Experience and training	What experience or training did the researcher have?	
Relationship with participants		
6. Relationship established	Was a relationship established prior to study commencement?	N/A
7. Participant knowledge of the interviewer	What did the participants know about the researcher? e.g. personal goals, reasons for doing the research?	Page 6
8. Interviewer characteristics	What characteristics were reported about the interviewer/facilitator? e.g. Bias, assumptions, reasons and interests in the research topic	Page 6
*Domain 2: study design*		
Theoretical framework		
9. Methodological orientationand Theory	What methodological orientation was stated to underpin the study? e.g. grounded theory, discourse analysis, ethnography, phenomenology, content analysis	Page 4–5
Participant selection		
10. Sampling	How were participants selected? e.g., purposive, convenience, consecutive, snowball	Page 4–6
11. Method of approach	How were participants approached? e.g., face‐to‐face, telephone, mail, email	Page 4–6
12. Sample size	How many participants were in the study?	Page 8
13. Non‐participation Setting	How many people refused to participate or dropped out? Reasons?	N/A ‐ none
14. Setting of data collection	Where was the data collected? e.g., home, clinic, workplace	Page 6
15. Presence of nonparticipants	Was anyone else present besides the participants and researchers?	Page 6 – no
16. Description of sample	What are the important characteristics of the sample? e.g. demographic data, date	Page 9–10
Data collection		
17. Interview guide	Were questions, prompts, and guides provided by the authors? Was it pilot tested?	Page 5 & Appendix 1
18. Repeat interviews	Were repeat interviews carried out? If yes, how many?	N/A
19. Audio/visual recording	Did the research use audio or visual recording to collect the data?	Page 7
20. Field notes	Were field notes made during and/or after the interview or focus group?	N/A
21. Duration	What was the duration of the interviews or focus group?	Pages 5, 8
22. Data saturation	Was data saturation discussed?	Page 8
23. Transcripts returned	Were transcripts returned to participants for comment and/or correction?	N/A
*Domain 3: analysis and findings*		
Data analysis		
24. Number of data coders	How many data coders coded the data?	Page 7
25. Description of the coding tree	Did the authors provide a description of the coding tree?	N/A
26. Derivation of themes	Were themes identified in advance or derived from the data?	Page 7
27. Software	What software, if applicable, was used to manage the data?	N/A
28. Participant checking	Did participants provide feedback on the findings?	Page 7 & 8
Reporting		
29. Quotations presented	Were participant quotations presented to illustrate the themes/findings? Was each quotation identified? e.g., participant number	Page 13–18
30. Data and findings consistent	Was there consistency between the data presented and the findings?	Page 13–18
31. Clarity of major themes	Were major themes clearly presented in the findings?	Page 13–18
32. Clarity of minor themes	Is there a description of diverse cases or a discussion of minor themes?	Page 13–18

## Appendix 2 Workshop content and timeline

**Table:**

Workshop #	Group	Content overview	Details
1 – March 2023	Consumers	Introductions/warm‐up	Introductions, housekeeping Previous experience with cancer/cancer screening
		Co‐design	Introduction to co‐design process
		Lung cancer and lung cancer screening (LCS)	Lung cancer overview Lung cancer screening overview Australian lung cancer screening program
		Recap of info/concepts	Recap of lung cancer screening
		Decision‐making for LCS	Benefits and harms of screening What would you want to know about lung cancer screening before deciding about participation?
		Decision tools	Introduction to decision support tools Explore examples of decision tools Explore what a decision tool might look like for lung cancer screening Explore how the tool could be used, what format, how reach people
2 – March 2023	Experts/clinicians	Introductions/warm‐up	Introductions, housekeeping Previous experience with cancer/cancer screening
		Co‐design	Introduction to co‐design process
		Decision‐making for LCS	What do patients need to know about lung cancer screening before deciding about participation?
		Decision support tools	Introduction/examples of decision support tools Explore what a decision tool might look like for lung cancer screening Explore how the tool could be used, what format, how reach people
Design/mock‐up of decision tools			
3 – June 2023	Consumers	Shared decision‐making*	Why shared‐decision‐making is important What it looks like in lung cancer screening How decision tools support shared decision‐making
		Revision of tools designed	Present concepts and visuals of the decision tools Feedback on 3x decision tools developed
4 – August 2023	Experts/clinicians	Revision of tools designed	Present concepts and visuals of the decision tools Feedback on 3x decision tools developed
5 – February 2024	Aboriginal and Torres Strait Islander peoples	Introductions/warm‐up	Introductions, housekeeping Previous experience with cancer/cancer screening
		Co‐design	Introduction to co‐design process
		Lung cancer and LCS	Lung cancer overview Lung cancer screening overview Australian lung cancer screening program
		Decision‐making for LCS	Benefits and harms of screening What would you want to know about lung cancer screening before deciding about participation?
		Shared decision‐making*	Why shared‐decision‐making is important What it looks like in lung cancer screening How decision tools support shared decision‐making
		Decision tools	Introduction to decision support tools Explore examples of decision tools Explore what a decision tool might look like for lung cancer screening Explore how the tool could be used, what format, how reach people
6 – April 2024	Aboriginal and Torres Strait Islander peoples	Introductions/warm‐up	Introductions, housekeeping Previous experience with cancer/cancer screening
		Co‐design	Introduction to co‐design process
		Lung cancer and LCS	Lung cancer overview Lung cancer screening overview Australian lung cancer screening program
		Decision‐making for LCS	Benefits and harms of screening What would you want to know about lung cancer screening before deciding about participation?
		Shared decision‐making*	Why shared‐decision‐making is important What it looks like in lung cancer screening How decision tools support shared decision‐making
		Decision tools	Introduction to decision support tools Explore examples of decision tools Explore what a decision tool might look like for lung cancer screening Explore how the tool could be used, what format, how reach people
Revision of tools			
Tools sent via email to workshop groups for further feedback.			

*Shared decision‐making content added based on Workshop 1 with consumers as we felt there was a gap in understanding and content covered in earlier workshops regarding shared decision‐making and the need to present balanced information to allow people to make informed decisions about LCS.

## Appendix 3 IPDAS Minimum Standards Regarding PDAs for Screening Tests^a^

**Table:**

	Yes	No
To Be Defined as a PDA, the PDA.		
1. Describes the health condition or problem for which the index decision is required.	x	
2. Identifies the target user.	x	
3. States explicitly the decision that must be considered.	x	
4. Describes the options available for index decision.	x	
5. Describes the positive features (i.e., benefits or advantages) of each option.	x	
6. Describes the negative features (i.e., harms, side effects, disadvantages) of each option.	x	
7. Helps patients clarify their values for outcomes of options by asking people to think about which positive and negative features of the options matter most to them and/or describing each option to help patients imagine the physical, social, and/or psychological effect.	x	
To Lower the Risk of Making a Biased Decision, the PDA…		
1. Shows the negative and positive features of the options with equal detail (eg, using similar fonts, sequence, presentation of statistical information).	x	
2. Provides information about the levels of uncertainty around event or outcome probabilities (eg, by giving a range or by using phrases such as ‘our best estimate is’).	x	
3. Describes what the screening test is designed to measure.	x	
4. Describes the next steps typically taken if the screening test detects the condition or problem.	x	
5. Describes the next steps if the condition or problem is not detected with the screening test.	x	
6. Has information about detection and treatment of disease that would not have caused problems if screening had not been done.	x	
7. Provides a production or publication date.^b^		x
8. Provides information about the funding source used for development.^b^	x	
9. Provides citations for scientific evidence selected.^b^	x	
10. Provides information about the update policy.^b^		x

Abbreviations: IPDAS, International Patient Decision Aid Standards; PDAs, patient decision aids.

a IPDAS also provides additional criteria for appraising quality of PDAs.

b Could be reported in a technical document accompanying the PDA.

## Appendix 4 Key information priorities for inclusion in decision support tools about lung cancer screening from workshops with consumers, healthcare experts and Aboriginal and Torres Strait Islander peoples

**Table:**

Key priority	Who	Details	Supporting quotes
Low‐dose computed tomography (LDCT) scan process	Consumers, including Aboriginal and Torres Strait Islander peoples	What happens on the day of the scan Scan location How people get there How long the scan takes How the scan is booked and organised What to wear to the scan Whether they would need to change clothes Availability of female technicians Cost of the scan and frequency	*The locations … the ease of the process and the steps to take [showing] how you book and organise something. (Consumer)*
	Steering committee	Information about ineligibility	*I wonder if a decision aid should have something in it that helps people understand why they might not get across the line and how to think about that? (Steering committee member)*
	Consumers	Accessibility (e.g., for those who might be living with a disability, whether a support person can be taken to the scan, and whether there were any preparation requirements).	*Location accessibility … how easy is it to get there? Is there public transport, parking … facilities to help? (Consumer)* *[If someone is from a] different cultural background and doesn't speak English as well, [they need] to know that they can bring their family member along as an advocate (Consumer)*
What happens after the scan	Consumers, including Aboriginal and Torres Strait Islander peoples	Potential scan results Treatment options Side effects compromising quality of life or financially Reassurance of options if something is found	*Some of this is about being reassured that there's a process, a suitable appropriate process afterwards to support us if we are diagnosed. (Consumer)*
	Steering committee	Commonness of lung nodules	*People may want to know what a positive test means. And how likely it is actually that it's going to tell me I've actually got lung cancer. And if it might do me any harm. (Steering committee member)*
What are the benefits of screening	Consumers, including Aboriginal and Torres Strait Islander peoples	Benefits of lung cancer screening are, being early detection (‘earlier the better’) Reluctant to present downsides or harms of lung cancer screening (LCS) to avoid deterring people (the perception was that the benefits (not dying from lung cancer) far outweighed any potential harms).	*[discussing harms of LCS] Yeah, but … the other option is you get lung cancer. So, it's just a no brainer … [harms are] irrelevant in my perception. (Consumer)* *I think some people might be concerned about going through that scan… I think I've read there's a low dose radiation, like an X ray. Well, look if it's picked up early, there's the high benefit of recovery. (Consumer)* *Taking everything up early, and then we don't have to worry about it. If there's a problem, we can catch it early. That's the way of looking at it rather than ‘Ohh I don't wanna [screen]’ … Of course, we don't wanna … but that's how I see it now and I'll say to my children, ‘That's what I owe you’. (Consumer)*
	Consumers, including Aboriginal and Torres Strait Islander peoples	Focus on ‘getting them in the door’ Rest of the information being on a need‐to‐know basis Express positivity about screening Cautious of the amount of potentially discouraging information. Reframe potential harms of screening as ‘other things to consider’	*Much bigger debate about informed choice in screening programmes in general, as opposed to programmes where we think from our public health point of view that we should be recommending people participate and actually the drivers around participation and slightly less around informed choice. And therefore, do you want a booklet that is actually promoting participation? Because we think on balance, most people will benefit more than they will risk being harmed as opposed to something like PSA testing where we think that the decision is much more of one around personal preferences. (Steering committee member)*
The need to manage stigma and discrimination in any resources.	Consumers	Ways to reduce the impact of stigma	*[regarding attending LCS] It's good to know no one is …. going to give me a hard time at the lung health check. (Consumer)*
	Consumers	How stigma and discrimination may impact uptake of screening in different regions due to the size of the communities.	*[In regional areas] we have even bigger stigma because the GP is gonna tell his wife and she's gonna tell the butcher's wife and she's gonna tell the… You know, then it's all around the town of 28 people. (Consumer)*
	Steering committee	Need to gain the consumer perspective on the use of the word cancer in the context of stigma. Suggestion to reduce stigma ‐ online and accessible tool so that people can view the information in private.	*Lung cancer being more highly stigmatised, you know, I think we do need to understand a bit more about the acceptability of using that language. (Steering committee member)*
Importance of tailoring for different language groups	Consumers	Culturally adapted information available for people who speak different languages.	*Access is dependent on the people understanding what it's all about and being able to access it without… Well, you don't need to have an interpreter standing next to you while you're getting your lung tested. (Consumer)*
	Consumers, including Aboriginal and Torres Strait Islander peoples	Beyond just language ‐ differences among communities and attitudes and how this would impact uptake of screening.	*I live in a very low social community, economic area, we have a lot of people that intergenerational welfare recipients, and they often have a fatalistic attitude. (Consumer)*
	Consumers, including Aboriginal and Torres Strait Islander peoples	Resource design	*[regarding images used in the decision support tools] in our region they would be faces of people would recognise in their own community. (Consumer)*
	Consumers, including Aboriginal and Torres Strait Islander peoples	The environment/clinic location (particularly for certain cultural communities)	*One area is [important for] any screening process is that it is provided in a non‐threatening environment. There is certain issue with the Southeast Asian community that doesn't want to get tested. But in … a public information session … we had 156 people that day, 97 people actually took the test. They were in the environment where [they] did not [have to] go into hospital. (Consumer)*
Cultural safety	Aboriginal and Torres Strait Islander peoples	Having an Aboriginal and Torres Strait Islander support worker present was considered important. Lack of cultural safety a key barrier, as well as cost, time, travel and the psychological impact. Acknowledged local cancer centre with no Aboriginal and Torres Strait Islander Health Workers and not culturally safe.	*The Cancer Centre here has no Black workers so there's no cultural… there's not a lot of cultural safety there unless you you've got a worker with you to walk you through the process. (Consumer)*
	Aboriginal and Torres Strait Islander peoples	For other cancer screening programmes, group sessions have been arranged and people like this, as they want to go together. Participants gave examples of groups ‘Aunty Jean's' which have been set up for Elders and people with chronic illnesses run by the Aboriginal Health team at the local health district, as well as Women's and Men's groups set up at health clinics.	*Well, they have the information, they cook a meal together, do some exercises and they get the blood pressure. (Consumer)*
